# Adjunctive diagnostic utility of POU2F3 in predominantly pulmonary high-grade neuroendocrine carcinomas with limited or discordant conventional marker expression: a case-control study

**DOI:** 10.3389/fmed.2026.1886172

**Published:** 2026-09-08

**Authors:** Xiangxiang Hu, Xiaoqiu Wang, Chuanying Li

**Affiliations:** 1 Department of Pathology, Jiashan Hospital of Traditional Chinese Medicine, Jiaxing City, Zhejiang Province, China; 2 Department of Pathology, Wuxi Branch of Ruijin Hospital, Shanghai Jiao Tong University School of Medicine, Wuxi, Jiangsu, China; 3 Department of Ruijin Hospital, Shanghai Jiao Tong University School of Medicine, Shanghai, China

**Keywords:** chromogranin A, diagnostic marker, large cell neuroendocrine carcinoma, neuroendocrine carcinoma, POU2F3 immunohistochemistry, small cell lung carcinoma

## Abstract

**Background:**

The diagnosis of high-grade neuroendocrine carcinoma may be challenging when conventional neuroendocrine markers show limited or discordant expression. POU2F3 is associated with a neuroendocrine-low subset of small cell carcinoma, but its incremental contribution in routine diagnostic practice remains incompletely defined.

**Objective:**

To evaluate the adjunctive diagnostic utility of POU2F3 in predominantly pulmonary high-grade neuroendocrine carcinomas, particularly in cases with limited or discordant conventional marker expression.

**Methods:**

This retrospective case-control study included 140 neuroendocrine carcinomas and 100 poorly differentiated non-neuroendocrine controls. POU2F3 expression was assessed in biopsy and resection specimens and analyzed according to histological subtype, chromogranin A status, marker-discordant status, and diagnostic-panel interpretation. Diagnostic performance was reported with 95% confidence intervals, and paired changes after incorporation of POU2F3 were evaluated using the exact McNemar test.

**Results:**

POU2F3 was positive in 28 of 140 neuroendocrine carcinomas and 2 of 100 controls. Positivity was more frequent in chromogranin A-negative/low than in chromogranin A-positive neuroendocrine carcinomas (34.5% vs. 10.6%; odds ratio, 4.46; 95% confidence interval, 1.84–10.82; *P* = 0.001). Positivity was identical in biopsy and resection specimens (20.0% vs. 20.0%; *P* = 1.000). POU2F3 alone showed 20.0% sensitivity and 98.0% specificity. Incorporation of POU2F3 increased panel sensitivity from 80.7% to 91.4% and accuracy from 87.1% to 93.3%, while specificity remained 96.0% (exact McNemar *P* < 0.001).

**Conclusion:**

POU2F3 has limited stand-alone sensitivity but provides incremental diagnostic support in selected neuroendocrine carcinomas with limited or discordant conventional marker expression. It should be used selectively and interpreted together with morphology and established neuroendocrine markers.

## 1 Introduction

Small cell lung carcinoma (SCLC) and large cell neuroendocrine carcinoma (LCNEC) are high-grade neuroendocrine carcinomas (*N*ECs) characterized by aggressive clinical behavior, rapid disease progression, and poor survival outcomes (1, 2). In routine diagnostic pathology, the recognition of NEC depends on the integration of morphology and immunohistochemistry, with chromogranin A (CgA), synaptophysin (SYN), CD56 (cluster of differentiation 56), and, increasingly, INSM1 (insulinoma-associated protein 1) serving as commonly used neuroendocrine markers (3–5). However, conventional marker expression is not always uniform. In particular, absent or reduced CgA expression may complicate the diagnosis of poorly differentiated tumors, especially in small biopsy specimens or in cases with overlapping morphology between NECs and non-neuroendocrine mimics (6, 7).

Recent transcription factor-based classifications have refined the biological understanding of SCLC and related high-grade NECs. SCLC can be divided into molecularly defined subgroups associated with ASCL1, NEUROD1, POU2F3, and YAP1 expression, with INSM1 and YAP1-related patterns also contributing to the recognition of neuroendocrine and non-neuroendocrine transcriptional states (8, 9). Among these, POU2F3-positive SCLC has been associated with a tuft-cell-like phenotype and relatively low expression of conventional neuroendocrine markers (10, 11). Mechanistic studies have further shown that POU2F3 participates in tuft-cell transcriptional programs and may be linked to POU2AF2-associated transcriptional regulation and mammalian SWI SNF chromatin-remodeling dependency (12–14). These findings suggest that POU2F3 is not merely a lineage-associated transcription factor but may also represent a recognizable immunophenotypic feature in a subset of neuroendocrine-low tumors.

The potential diagnostic relevance of POU2F3 has been reported mainly in neuroendocrine-low or neuroendocrine-negative SCLC. Baine et al. showed that POU2F3 immunohistochemistry may help identify SCLC with absent or minimal conventional neuroendocrine marker expression, and subsequent studies further supported its value in selected SCLC and LCNEC contexts (10, 15, 16). A larger immunohistochemical study further supported POU2F3 as a diagnostic marker for neuroendocrine-low or neuroendocrine-negative SCLC, while also showing rare expression in selected non-SCLC tumors, emphasizing the need for panel-based interpretation (15). Nevertheless, its role in routine diagnostic settings remains incompletely defined. Existing studies have focused largely on SCLC-P biology, although emerging data have extended POU2F3-related classification to LCNEC and extrapulmonary NECs. However, fewer data are available regarding its practical value in distinguishing NECs from poorly differentiated non-neuroendocrine mimics or in resolving cases with absent, low, or discordant conventional neuroendocrine marker expression (17–19).

This distinction is clinically relevant because CgA-negative and CgA-low NECs may overlap morphologically and immunophenotypically with poorly differentiated squamous cell carcinoma, basaloid squamous cell carcinoma, poorly differentiated adenocarcinoma, or other high-grade mimics. In such settings, the diagnostic question is not whether POU2F3 can replace conventional neuroendocrine markers, but whether it can provide additional support when the conventional immunohistochemical profile is incomplete or discordant. Therefore, POU2F3 should be evaluated within a broader diagnostic panel and interpreted together with morphology, CgA, SYN, CD56, and INSM1.

In this study, POU2F3 immunohistochemistry was evaluated in 240 cases, including 140 NECs and 100 non-NEC controls. The aims were to determine the distribution of POU2F3 expression across NECs and non-NEC controls, to assess whether POU2F3 positivity is enriched in SCLC and in CgA-negative or CgA-low NECs, and to evaluate whether incorporation of POU2F3 into a conventional neuroendocrine immunohistochemical panel improves diagnostic support, particularly in marker-discordant cases.

## 2 Materials and methods

### 2.1 Case selection

This retrospective study included formalin-fixed, paraffin-embedded tumor specimens consecutively retrieved from the institutional pathology archive between May 2024 and May 2026. All eligible high-grade neuroendocrine carcinoma cases meeting the study criteria were included. Control cases were consecutively selected within diagnostic categories to represent the principal poorly differentiated non-neuroendocrine tumors encountered in the differential diagnosis of high-grade neuroendocrine carcinoma.

Only one specimen per patient was included. When more than one specimen was available, the earliest pretreatment specimen containing sufficient viable tumor tissue for complete morphological and immunohistochemical assessment was selected. Eligible cases were required to have: (1) a final pathological diagnosis of high-grade neuroendocrine carcinoma or a selected poorly differentiated non-neuroendocrine mimic; (2) sufficient viable tumor tissue for morphological reassessment and completion of the conventional immunohistochemical panel and POU2F3 staining; and (3) complete clinicopathological and immunohistochemical data. Specimens were excluded if viable tumor tissue was insufficient, extensive necrosis or crush artifact prevented reliable reassessment, immunohistochemical staining was incomplete or technically uninterpretable, or the specimen represented duplicate sampling from the same patient.

The neuroendocrine carcinoma cohort comprised 90 small cell lung carcinomas, 35 large cell neuroendocrine carcinomas, and 15 other high-grade neuroendocrine carcinomas. The control cohort comprised 35 poorly differentiated squamous cell carcinomas, 35 poorly differentiated adenocarcinomas, 20 basaloid squamous cell carcinomas, and 10 other poorly differentiated non-neuroendocrine tumors. These control categories were selected because they represent clinically relevant morphological or immunophenotypic mimics of high-grade neuroendocrine carcinoma.

The reference diagnosis had been established before the present study through integrated evaluation of tumor morphology, anatomical site, and routine immunohistochemical findings according to the applicable World Health Organization classification criteria. POU2F3 was not part of the original reference diagnosis. All cases were subsequently reviewed by two pathologists to confirm diagnostic eligibility and adequacy of tumor tissue for study inclusion.

### 2.2 Immunohistochemistry and scoring

All immunohistochemical staining was performed on 4-*μ*m-thick formalin-fixed, paraffin-embedded sections using laboratory-validated protocols on an automated immunostaining platform. The primary antibodies included POU2F3 (mouse monoclonal, clone 1091214, catalog no. MAB11625, 5 μg/mL; R&D Systems, a Bio-Techne brand, Minneapolis, MN, USA), chromogranin A (CgA; mouse monoclonal, clone 948128, catalog no. MAB90981, 5 μg/mL; R&D Systems), synaptophysin (SYN; mouse monoclonal, clone 959904, catalog no. MAB5555, 15 μg/mL; R&D Systems), CD56/NCAM1 (mouse monoclonal, clone 301021, catalog no. MAB24081, 25 μg/mL; R&D Systems), and INSM1 (mouse monoclonal, clone A-8, catalog no. sc-271408, 1:250; Santa Cruz Biotechnology, Dallas, TX, USA).

Heat-induced epitope retrieval was performed using a laboratory-validated retrieval protocol. POU2F3 and CD56 were incubated for 1 h at room temperature, whereas CgA, SYN, and INSM1 were incubated overnight at 4 °C. Immunoreactivity was detected using a horseradish peroxidase polymer-based system with diaminobenzidine as the chromogen, followed by hematoxylin counterstaining. Appropriate external positive-control tissues were included in each staining run. Negative reagent controls were prepared by omitting the primary antibody. Detailed antibody and staining conditions are provided in Supplementary Table S6.

The percentage of positively stained tumor cells and nuclear staining intensity were independently assessed by two pathologists. Staining intensity was categorized as weak, moderate, or strong. POU2F3 positivity was defined as nuclear staining in at least 10% of tumor cells. For descriptive analyses, staining extent based on the percentage of positive tumor cells was categorized as negative (<10%), low (10%–25%), intermediate (26%–50%), or high (>50%).

For the purposes of this study, CgA expression was operationally categorized as absent (0%), low (1%–10%), or positive (>10%). This classification was used to distinguish complete absence from focal low-level expression and was not intended to represent a universally validated biological threshold. SYN, CD56, and INSM1 were classified as positive or negative according to routine diagnostic interpretation. POU2F3 staining percentage and extent were independently assessed by two pathologists using the corresponding hematoxylin and eosin sections to identify viable tumor. Discrepancies were resolved by joint review and consensus.

### 2.3 Diagnostic definitions

The conventional immunohistochemical panel comprised CgA, SYN, CD56, and INSM1. Panel interpretation was categorized as supportive, equivocal, or non-supportive for neuroendocrine differentiation in the corresponding morphological context. A supportive result indicated sufficient integrated immunohistochemical evidence for neuroendocrine differentiation. An equivocal result indicated incomplete or conflicting immunohistochemical evidence, whereas a non-supportive result indicated absence of sufficient immunohistochemical support.

Marker-discordant cases were defined as morphologically suspected neuroendocrine carcinomas showing one or more of the following: (1) absent or low CgA expression; (2) discordant expression among CgA, SYN, CD56, and INSM1; or (3) an equivocal conventional-panel interpretation despite compatible morphology. Marker discordance did not necessarily indicate a non-supportive panel interpretation, because some tumors retained sufficient overall evidence for neuroendocrine differentiation despite an atypical marker combination.

POU2F3 added diagnostic value was defined as a change from an equivocal or non-supportive conventional-panel interpretation to a supportive interpretation after POU2F3 was incorporated into the integrated panel assessment. For diagnostic-performance analyses, supportive interpretations were classified as positive, whereas equivocal and non-supportive interpretations were classified as negative. Diagnostic performance was evaluated for POU2F3 alone, the conventional panel, and the conventional panel combined with POU2F3.

The final pathological diagnosis was used as the reference standard. Because the conventional neuroendocrine markers had contributed to the original pathological diagnosis, complete independence between the conventional panel and the reference standard could not be ensured. POU2F3, however, had not been used in establishing the original reference diagnosis.

### 2.4 Statistical analysis

Continuous variables were summarized as mean and standard deviation or median and interquartile range, as appropriate. Categorical variables were summarized as numbers and percentages and compared using the chi-square test, Fisher's exact test, or the Fisher–Freeman–Halton exact test. The percentage of POU2F3-positive tumor cells in biopsy and resection specimens was compared using the Mann–Whitney U test.

Sensitivity, specificity, positive predictive value, negative predictive value, and accuracy were reported as point estimates with Wilson 95% confidence intervals. Positive and negative likelihood ratios were calculated with logarithm-based 95% confidence intervals. Paired changes between the conventional panel and the panel incorporating POU2F3 were evaluated using the exact McNemar test. Receiver operating characteristic analysis based on the percentage of POU2F3-positive tumor cells was performed as an exploratory analysis. The 95% confidence interval for the area under the receiver operating characteristic curve was estimated using stratified bootstrap resampling with 5,000 iterations. Threshold analyses at 5%, 10%, and 15% were considered exploratory.

All eligible neuroendocrine carcinoma cases meeting the study criteria during the study period were included. Analyses involving LCNEC and other neuroendocrine carcinomas were considered exploratory because of limited subgroup size. All tests were two-sided, and *P* values less than 0.05 were considered statistically significant. Statistical analyses were performed using IBM SPSS Statistics, (version 29.0.1.0.171, IBM Corp., Armonk, NY, USA).

## 3 Results

### 3.1 Study cohort

**Figure 1:**
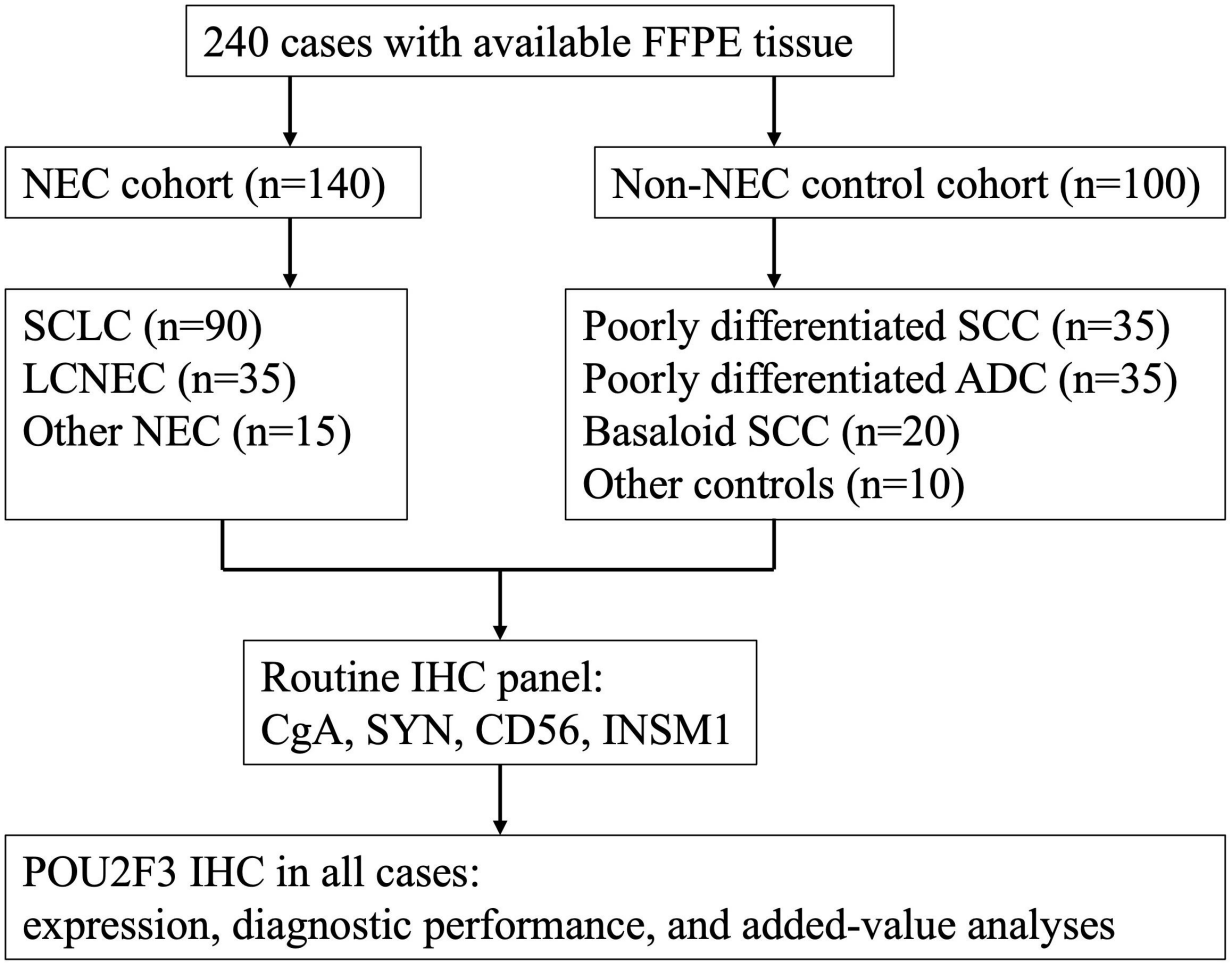
Study cohort composition and analytical workflow.

A total of 240 cases were included in the study, comprising 140 NECs and 100 non-NEC controls. The case selection process and analytical workflow are shown in Figure 1. Age, sex, and specimen type were comparable between the two groups. In contrast, significantly higher expression rates of SYN, CD56, INSM1, and POU2F3 were observed in NECs than in non-NEC controls, with *P* < 0.001 (Table 1). These findings supported comparison of conventional neuroendocrine markers and POU2F3 between NECs and non-NEC mimics.

**Table 1:** Clinicopathological characteristics of the study cohort.

Variable	Overall (*n* = 240)	NEC (*n* = 140)	Non-NEC controls (*n* = 100)	*P*-value
Age, years	63.7 ± 9.9	63.0 ± 10.0	64.8 ± 9.7	0.146
Sex				0.345
Male	149 (62.1%)	83 (59.3%)	66 (66.0%)	
Female	91 (37.9%)	57 (40.7%)	34 (34.0%)	
Specimen type				0.152
Biopsy	119 (49.6%)	75 (53.6%)	44 (44.0%)	
Resection	121 (50.4%)	65 (46.4%)	56 (56.0%)	
Primary site				0.051
Lung	169 (70.4%)	106 (75.7%)	63 (63.0%)	
Esophagus	30 (12.5%)	14 (10.0%)	16 (16.0%)	
Mediastinum	9 (3.8%)	6 (4.3%)	3 (3.0%)	
Gastrointestinal tract	22 (9.2%)	12 (8.6%)	10 (10.0%)	
Other	10 (4.2%)	2 (1.4%)	8 (8.0%)	
Histological subtype				NA
SCLC	90 (37.5%)	90 (64.3%)	0 (0.0%)	
LCNEC	35 (14.6%)	35 (25.0%)	0 (0.0%)	
Other NEC	15 (6.2%)	15 (10.7%)	0 (0.0%)	
Poorly differentiated SCC	35 (14.6%)	0 (0.0%)	35 (35.0%)	
Poorly differentiated ADC	35 (14.6%)	0 (0.0%)	35 (35.0%)	
Basaloid SCC	20 (8.3%)	0 (0.0%)	20 (20.0%)	
Other non-NEC control	10 (4.2%)	0 (0.0%)	10 (10.0%)	
SYN positive	117 (48.8%)	113 (80.7%)	4 (4.0%)	<0.001
CD56 positive	132 (55.0%)	118 (84.3%)	14 (14.0%)	<0.001
INSM1 positive	130 (54.2%)	128 (91.4%)	2 (2.0%)	<0.001
POU2F3 positive	30 (12.5%)	28 (20.0%)	2 (2.0%)	<0.001

Values are mean ± standard deviation or *n* (%). Age was compared using Welch's *t* test; sex and specimen type using Fisher's exact test; primary site using the chi-square test.

NA, not applicable.

Among the 140 NECs, 75 were biopsy specimens and 65 were resection specimens. POU2F3 positivity was identical in biopsy and resection specimens (*P* = 1.000). No significant differences were observed in staining-extent distribution, marker-discordant status, added diagnostic value, or supportive panel interpretation. Among POU2F3-positive cases, the median percentage of positive tumor cells was 25.0% in biopsies and 40.0% in resections (*P* = 0.085; Table 2).

**Table 2:** POU2F3 results and diagnostic-panel interpretations in NECs according to specimen type.

Variable	Biopsy (*n* = 75)	Resection (*n* = 65)	*P*-value
POU2F3 positive	15 (20.0%)	13 (20.0%)	1.000
POU2F3 intensity			0.671
Negative	60 (80.0%)	52 (80.0%)	
Weak	8 (10.7%)	4 (6.2%)	
Moderate	6 (8.0%)	7 (10.8%)	
Strong	1 (1.3%)	2 (3.1%)	
POU2F3-positive tumor cells among positive cases, median (IQR)	25.0% (15.0%–37.5%)	40.0% (20.0%–50.0%)	0.085
Marker-discordant	23 (30.7%)	17 (26.2%)	0.579
POU2F3 added diagnostic value	7 (9.3%)	8 (12.3%)	0.595
Supportive conventional panel	62 (82.7%)	51 (78.5%)	0.668
Supportive conventional panel + POU2F3	69 (92.0%)	59 (90.8%)	1.000

Categorical variables were compared using Fisher's exact test or the Fisher-Freeman-Halton exact test, as appropriate. The percentage of POU2F3-positive tumor cells among positive cases was compared using the Mann–Whitney *U*-test. The same 10% positivity threshold was applied to biopsy and resection specimens.

### 3.2 Representative POU2F3 immunohistochemical patterns

**Figure 2:**
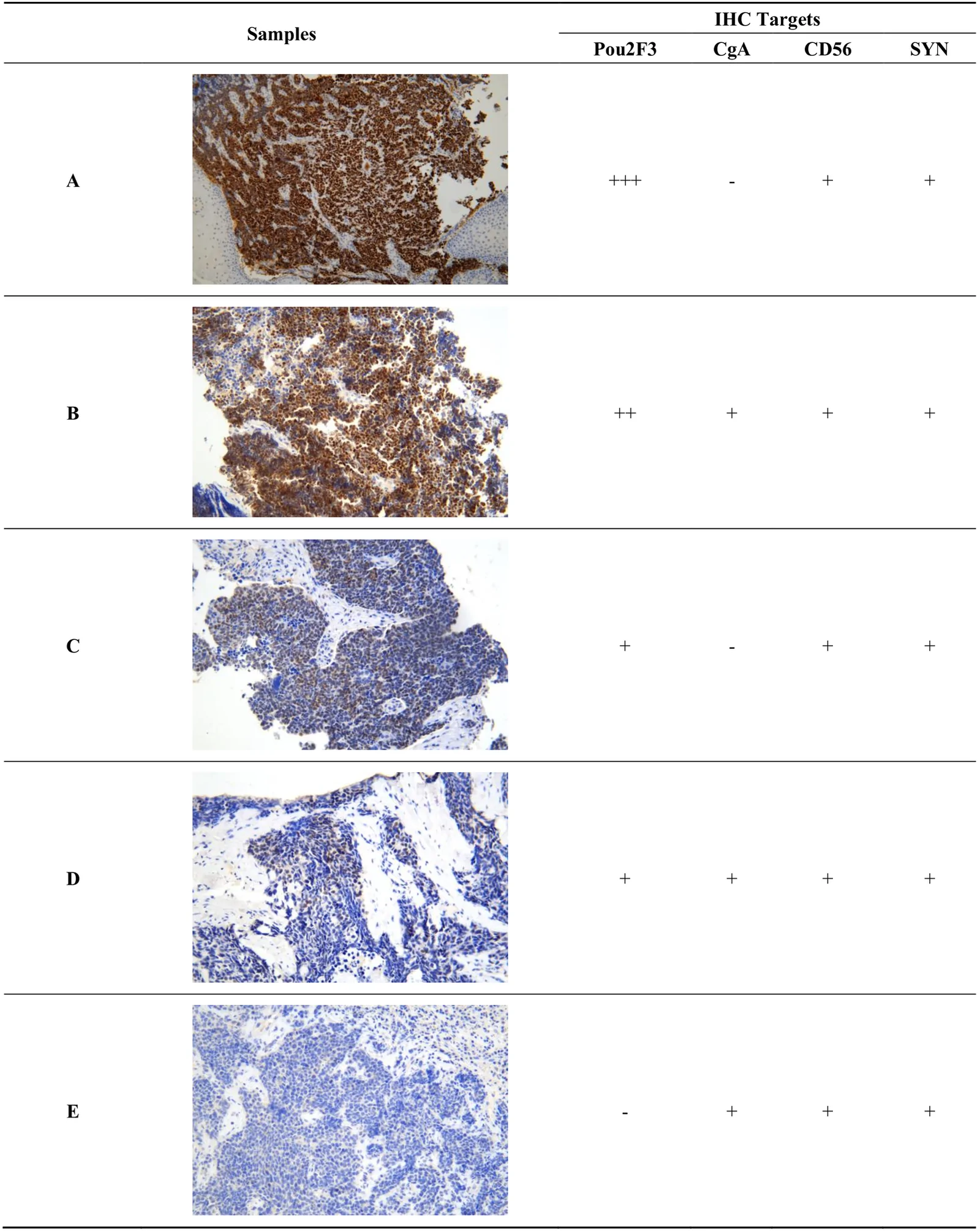
Representative POU2F3 immunohistochemical staining patterns and marker profiles in selected NEC cases. **(A)** Strong nuclear POU2F3 positivity (+++) in esophageal small cell carcinoma (100×). **(B)** Moderate nuclear POU2F3 positivity (++) in lung small cell carcinoma (200×). **(C)** Weak nuclear POU2F3 positivity (+) in lung small cell carcinoma (200×). **(D)** Mediastinal mixed high-grade neuroendocrine carcinoma, predominantly composed of small cell carcinoma with a minor large cell neuroendocrine carcinoma component, showing weak nuclear POU2F3 positivity and positive expression of CgA, SYN, and CD56 (200×). **(E)** Representative POU2F3-negative NEC (200×).

Representative POU2F3 immunohistochemical staining patterns are shown in Figure 2. A spectrum of nuclear staining patterns was observed in the selected cases, including strong, moderate, weak, and negative staining. POU2F3 expression was observed in NECs with variable conventional neuroendocrine marker profiles. In the mediastinal mixed high-grade NEC shown in Figure 2D, weak POU2F3 nuclear positivity was accompanied by positive CgA, SYN, and CD56 expression.

### 3.3 POU2F3 expression by diagnostic group and histological subtype

POU2F3 was positive in 28 of 140 NECs and 2 of 100 non-NEC controls. Among NECs, positivity was observed in 24 of 90 SCLCs, 3 of 35 LCNECs, and 1 of 15 other NECs. The overall distribution differed across NEC subtypes (exact *P* = 0.031); however, the LCNEC and other NEC estimates had wide confidence intervals and were considered exploratory. Both POU2F3-positive non-NEC controls showed weak, low-percentage nuclear staining. Detailed subtype results are provided in Supplementary Table S1.

### 3.4 Association between CgA status and POU2F3 expression

POU2F3 positivity differed significantly among CgA-negative, CgA-low, and CgA-positive NECs (overall exact *P* = 0.002). When CgA-negative and CgA-low cases were combined, POU2F3 positivity was higher than in CgA-positive NECs (OR 4.46; 95% CI, 1.84–10.82; *P* = 0.001). Marker discordance and added diagnostic value were also more frequent in the CgA-negative/low group (*P* < 0.001; Table 3).

**Table 3:** Association of CgA expression status with POU2F3 positivity, marker discordance, and added diagnostic value in NECs.

CgA status in NEC	*n*	POU2F3 positive, *n* (%)	Marker-discordant, *n* (%)	POU2F3 added value, *n* (%)
CgA-negative	32	12 (37.5%)	19 (59.4%)	7 (21.9%)
CgA-low	23	7 (30.4%)	14 (60.9%)	6 (26.1%)
CgA-positive	85	9 (10.6%)	7 (8.2%)	2 (2.4%)
Overall exact *P* value		0.002	<0.001	<0.001
CgA-negative/low combined	55	19 (34.5%)	33 (60.0%)	13 (23.6%)
CgA-positive	85	9 (10.6%)	7 (8.2%)	2 (2.4%)

Overall three-group comparisons used the Fisher-Freeman-Halton exact test. For CgA-negative/low vs. CgA-positive NECs: POU2F3 positivity OR 4.46 (95% CI 1.84–10.82), *P* = 0.001; marker discordance OR 16.71 (95% CI 6.51–42.91), *P* < 0.001; added diagnostic value OR 12.85 (95% CI 2.77–59.58), *P* < 0.001.

### 3.5 Diagnostic performance of POU2F3 and combined immunohistochemical panels

**Figure 3:**
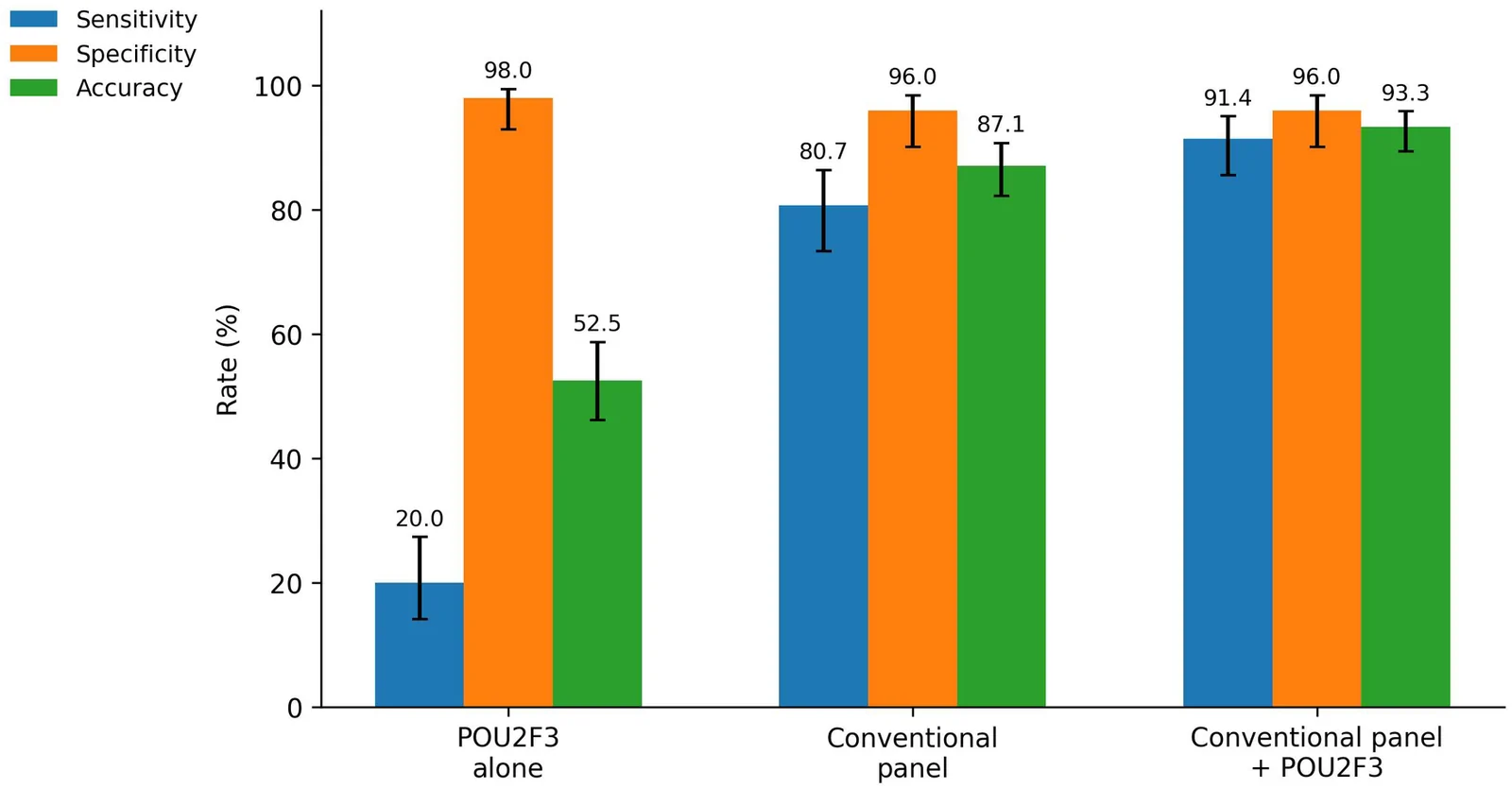
Diagnostic performance of POU2F3 alone, the conventional panel, and the conventional panel combined with POU2F3. Error bars indicate Wilson 95% confidence intervals. The exact McNemar test for supportive classifications in NECs showed a significant improvement after incorporation of POU2F3 (*P* < 0.001).

POU2F3 alone showed limited sensitivity of 20.0% (95% CI, 14.2%–27.4%) but high specificity of 98.0% (95% CI, 93.0%–99.4%). The conventional panel showed 80.7% sensitivity and 96.0% specificity. After incorporation of POU2F3, sensitivity increased to 91.4% and accuracy increased from 87.1% to 93.3%, while specificity remained 96.0%. The paired increase in supportive classifications was significant by the exact McNemar test (*P* < 0.001). The negative likelihood ratio decreased from 0.201 to 0.089 (Table 4 and Figure 3).

**Table 4:** Diagnostic performance of POU2F3 alone and combined immunohistochemical panels.

Diagnostic model	Sensitivity, % (95% CI)	Specificity, % (95% CI)	PPV, % (95% CI)	NPV, % (95% CI)	Accuracy, % (95% CI)	LR + (95% CI)	LR- (95% CI)
POU2F3 alone	20.0 (14.2–27.4)	98.0 (93.0–99.4)	93.3 (78.7–98.2)	46.7 (40.0–53.4)	52.5 (46.2–58.7)	10.00 (2.44–41.02)	0.816 (0.748–0.891)
Conventional panel	80.7 (73.4–86.4)	96.0 (90.2–98.4)	96.6 (91.5–98.7)	78.0 (69.9–84.5)	87.1 (82.2–90.7)	20.18 (7.70–52.89)	0.201 (0.143–0.283)
Conventional panel + POU2F3	91.4 (85.6–95.0)	96.0 (90.2–98.4)	97.0 (92.5–98.8)	88.9 (81.6–93.5)	93.3 (89.4–95.9)	22.86 (8.74–59.79)	0.089 (0.052–0.154)

Supportive interpretations were classified as positive, whereas equivocal and non-supportive interpretations were classified as negative. The paired difference between the conventional and combined panels was significant by the exact McNemar test (*P* < 0.001). PPV and NPV reflect the case-control sampling proportion and should not be directly extrapolated to unselected populations.

### 3.6 Added diagnostic value of POU2F3 in selected scenarios

POU2F3 provided additional diagnostic support in 15 of 140 NECs, corresponding to an absolute increase of 10.7 percentage points in supportive interpretations. The incremental contribution was greater in CgA-negative/low NECs (13/55, 23.6%) and marker-discordant NECs (15/40, 37.5%). No added value was observed in marker-concordant NECs, and no non-NEC control was reclassified as supportive after POU2F3 incorporation. Detailed results are provided in Supplementary Table S5.

## 4 Discussion

The principal finding of this study was not that POU2F3 functions as a broadly sensitive neuroendocrine marker, but that it provided selective incremental support in cases with limited or discordant conventional marker expression. POU2F3 alone showed only 20.0% sensitivity. However, its incorporation into the conventional panel increased sensitivity by 10.7 percentage points, from 80.7% to 91.4%, without reducing specificity. The incremental contribution was concentrated in marker-discordant and CgA-negative/low NECs.

CgA status was used as one clinically interpretable stratification variable, but it does not capture the complete neuroendocrine immunophenotype. SYN and INSM1 are generally more sensitive than CgA in poorly differentiated NECs, and some CgA-negative tumors remain readily recognizable because of retained SYN or INSM1 expression. Accordingly, the CgA-based analyses should be interpreted as secondary subgroup analyses, whereas marker discordance across the complete conventional panel provides a broader representation of diagnostically atypical cases.

POU2F3 positivity was identical in biopsy and resection specimens in the present cohort, and no significant specimen-type differences were observed in staining extent or incremental diagnostic support. Therefore, separate positivity thresholds were not applied. Nevertheless, a small biopsy may fail to capture spatially heterogeneous expression. A paired-sample study found significant correlation between POU2F3 expression in limited samples and corresponding resections, supporting the feasibility of biopsy-based assessment, whereas a recent large study also documented marked intratumoral POU2F3 heterogeneity in a small proportion of surgically resected SCLCs. These findings support use of the same interpretive threshold while maintaining caution regarding negative results in limited tissue.

Another methodological consideration is assay standardization. The antibody clones used in the present study were selected because they had been incorporated into the laboratory's validated immunohistochemical workflow and consistently produced appropriate control staining. Nevertheless, published studies have used different POU2F3 antibody clones, staining platforms, and scoring systems. For example, Baine et al. used clone 6D1 on the Leica Bond III platform and applied an H-score-based interpretation, whereas the present study used clone 1091214 and a percentage-based scoring approach (10). Differences in antibody clone, antigen retrieval, detection system, and scoring criteria may partly contribute to variability in reported POU2F3 positivity rates across studies. Direct numerical comparisons should therefore be interpreted cautiously, and multicenter validation using standardized staining and scoring protocols remains warranted.

Two non-NEC controls showed weak POU2F3 nuclear staining at low percentages. One was a poorly differentiated squamous cell carcinoma and the other was a basaloid squamous cell carcinoma. Neither was reclassified as supportive for NEC after integrated panel interpretation. These cases demonstrate that weak or focal POU2F3 staining is not entirely specific and should not override morphology or lineage markers. Previous studies have also identified POU2F3 expression in a small subset of squamous and basaloid squamous carcinomas.

The enrichment of POU2F3 expression in SCLC is consistent with previous molecular and immunohistochemical studies of SCLC subtypes. SCLC has been classified into transcription factor-associated subtypes defined by ASCL1, NEUROD1, POU2F3, and YAP1, and POU2F3-positive tumors have been linked to a tuft-cell-like or neuroendocrine-low phenotype (8, 10, 11). In the present cohort, POU2F3 positivity was concentrated mainly in SCLC, while lower positivity rates were observed in LCNEC and other NEC subtypes. Recent data on POU2F3-expressing LCNEC also suggest that a subset of LCNECs may share morphologic, immunohistochemical, and genomic features with POU2F3-associated SCLC, supporting the need to evaluate POU2F3 beyond conventional SCLC alone (20). Similar transcription factor-based subgrouping has also been described in extrapulmonary small cell or neuroendocrine carcinomas, suggesting that POU2F3-related phenotypes are not restricted to pulmonary SCLC (21). Because ASCL1, NEUROD1, YAP1, and genomic profiling were not systematically evaluated, POU2F3 positivity in this study should be regarded as an immunohistochemical phenotype rather than proof of SCLC-P classification.

The association between POU2F3 and reduced CgA expression is one of the most relevant findings of this study. Conventional neuroendocrine diagnosis relies heavily on markers such as CgA, SYN, CD56, and INSM1, but CgA-negative or CgA-low tumors may create diagnostic uncertainty, particularly in small biopsies or poorly differentiated tumors. Previous studies have suggested that POU2F3 may be useful in neuroendocrine-low or neuroendocrine-negative SCLC, especially when conventional neuroendocrine marker expression is absent or minimal (10, 15). In the present study, POU2F3 positivity was more frequently observed in CgA-negative and CgA-low NECs than in CgA-positive NECs. Marker-discordant status and added diagnostic value were also concentrated in these cases. These findings indicate that the main diagnostic relevance of POU2F3 lies not in routine marker-concordant NECs, but in tumors with incomplete or discordant conventional neuroendocrine marker profiles.

The diagnostic performance analysis further supports this interpretation. POU2F3 alone was characterized by high specificity but limited sensitivity, indicating that it is not suitable as a general screening marker for NEC. In contrast, the conventional immunohistochemical panel showed higher sensitivity and overall accuracy. When POU2F3 was added to the conventional panel, sensitivity and accuracy were further improved, while specificity was maintained. This pattern suggests that POU2F3 is most useful as an adjunctive component of a broader immunohistochemical strategy. The added-value analysis also showed that POU2F3 contributed mainly in CgA-negative, CgA-low, and marker-discordant NECs, while little or no additional value was observed in CgA-positive or marker-concordant NECs. Therefore, routine application of POU2F3 to all NECs may not be necessary, whereas selective use in diagnostically challenging cases appears more appropriate.

The present findings also have implications for the interpretation of POU2F3-positive non-NEC controls. Although POU2F3 positivity was rare in the control group and limited to weak staining, such findings emphasize that POU2F3 should not be interpreted in isolation. Morphology and the conventional neuroendocrine panel remain essential for diagnosis. This is especially relevant because CD56 may occasionally be expressed in non-neuroendocrine tumors, and weak or focal immunoreactivity can occur in poorly differentiated mimics. In this context, POU2F3 should be interpreted together with CgA, SYN, CD56, INSM1, and histological features. These findings support consideration of POU2F3 after routine marker evaluation, particularly when CgA expression is absent or reduced or when conventional marker results are discordant.

Mechanistically, POU2F3 has been described as a regulator of tuft-cell-like transcriptional programs in a subset of SCLC. Prior studies have linked POU2F3-driven tumors to POU2AF2-associated transcriptional regulation and mammalian SWI SNF chromatin-remodeling dependency (11, 12, 22, 23). These mechanistic findings provide biological support for the observed immunohistochemical phenotype. However, the present study was designed as a diagnostic pathology study rather than a mechanistic or therapeutic study. Therefore, therapeutic implications related to POU2F3 expression, including sensitivity to mammalian SWI SNF-targeted approaches, should be considered hypothesis-generating only. Clinical outcome data and treatment response data were not analyzed, and no conclusion regarding prognosis or therapeutic prediction can be drawn from this cohort.

Tumor heterogeneity should also be considered when interpreting POU2F3 staining. Previous work has shown that transcription factor-based subtype assignment may vary across tumor regions or between primary and metastatic sites, underscoring the need for comprehensive immunohistochemical evaluation (24, 25). In this study, POU2F3 staining extent varied across cases, and positivity was not uniformly observed in all CgA-negative or CgA-low NECs. This heterogeneity suggests that POU2F3 should be viewed as a marker of a selected biological phenotype rather than a universal marker for neuroendocrine differentiation. For mixed tumors or small biopsy specimens, adequate sampling and interpretation in the full morphologic and immunophenotypic context remain essential.

Several limitations should be acknowledged. First, this was a retrospective single-center case-control study, and inclusion required the availability of adequate archived tumor tissue. Second, the conventional neuroendocrine markers evaluated in the study had also contributed to the original pathological diagnosis; therefore, partial incorporation bias could not be completely excluded. Third, the NEC-to-control sampling ratio limits the direct generalizability of positive predictive value, negative predictive value, and accuracy to unselected clinical populations. Fourth, although biopsy and resection specimens showed similar results, limited tissue may not fully capture spatially heterogeneous POU2F3 expression. Fifth, the LCNEC and other NEC subgroups were small, and their estimates were exploratory and imprecise. Finally, external multicenter validation using harmonized antibody clones, staining platforms, antigen-retrieval conditions, detection systems, and scoring criteria is required.

## 5 Conclusions

In conclusion, POU2F3 immunohistochemistry showed limited sensitivity but high specificity for NEC diagnosis in this case-control cohort. POU2F3 positivity was enriched in SCLC and in NECs with absent or low CgA expression. When incorporated into a conventional neuroendocrine immunohistochemical panel, POU2F3 improved diagnostic sensitivity and overall accuracy while maintaining specificity. Its added diagnostic value was most evident in CgA-negative, CgA-low, and marker-discordant NECs, whereas limited additional value was observed in marker-concordant cases. These findings support the selective use of POU2F3 as an adjunctive diagnostic marker for diagnostically challenging NECs, rather than as a stand-alone neuroendocrine marker.

## Data Availability

The raw data supporting the conclusions of this article will be made available by the authors, without undue reservation.
