# Supplementary material for: Adjunctive diagnostic utility of POU2F3 in predominantly pulmonary high-grade neuroendocrine carcinomas with limited or discordant conventional marker expression: a case-control study
Source: Front Med (Lausanne). 2026 Sep 8;13:1886172. doi: 10.3389/fmed.2026.1886172 (PMC13593409; doi:10.3389/fmed.2026.1886172)
Supplement: Supplementary file 1 [file Table1.docx]

**Supplementary Materials**

**Table S1.** POU2F3 expression according to diagnostic group and histological subtype.

| **Diagnostic group/subtype** | **n** | **POU2F3 positive, n (%)** | **95% CI** | **Low (10%–25%)** | **Intermediate (26%–50%)** | **High (>50%)** |
| --- | --- | --- | --- | --- | --- | --- |
| NEC overall | 140 | 28 (20.0%) | 14.2-27.4% | 12 | 13 | 3 |
| SCLC | 90 | 24 (26.7%) | 18.6-36.6% | 11 | 11 | 2 |
| LCNEC | 35 | 3 (8.6%) | 3.0-22.4% | 1 | 2 | 0 |
| Other NEC | 15 | 1 (6.7%) | 1.2-29.8% | 0 | 0 | 1 |
| Non-NEC controls overall | 100 | 2 (2.0%) | 0.6-7.0% | 2 | 0 | 0 |
| Poorly differentiated SCC | 35 | 1 (2.9%) | 0.5-14.5% | 1 | 0 | 0 |
| Poorly differentiated ADC | 35 | 0 (0.0%) | 0.0-9.9% | 0 | 0 | 0 |
| Basaloid SCC | 20 | 1 (5.0%) | 0.9-23.6% | 1 | 0 | 0 |
| Other non-NEC control | 10 | 0 (0.0%) | 0.0-27.8% | 0 | 0 | 0 |

Confidence intervals are Wilson 95% confidence intervals. LCNEC and other NEC estimates are exploratory because of limited subgroup size.

**Table S2.** Clinicopathological and immunohistochemical features of POU2F3-positive non-NEC controls.

| **Case** | **Specimen/site** | **Final diagnosis** | **POU2F3** | **CgA** | **SYN** | **CD56** | **INSM1** | **Combined panel interpretation** |
| --- | --- | --- | --- | --- | --- | --- | --- | --- |
| A | Resection/Lung | Poorly differentiated SCC | 10%, weak | Negative | Negative | Negative | Negative | Non-supportive |
| B | Biopsy/Esophagus | Basaloid SCC | 12%, weak | Negative | Negative | Positive | Negative | Equivocal |

Case labels were anonymized to reduce re-identification risk. Both POU2F3-positive control results were weak and low-percentage, and neither case was reclassified as supportive for NEC.

**Table S3.** Exploratory sensitivity analysis of POU2F3 positivity thresholds.

| **Threshold for positivity** | **NEC positive, n/N (%)** | **Control positive, n/N (%)** | **Sensitivity, %** | **Specificity, %** | **Accuracy, %** | **Youden index** |
| --- | --- | --- | --- | --- | --- | --- |
| ≥5% | 28/140 (20.0%) | 2/100 (2.0%) | 20.0 | 98.0 | 52.5 | 0.180 |
| ≥10% | 28/140 (20.0%) | 2/100 (2.0%) | 20.0 | 98.0 | 52.5 | 0.180 |
| ≥15% | 27/140 (19.3%) | 0/100 (0.0%) | 19.3 | 100.0 | 52.9 | 0.193 |

No case showed 5%–9% POU2F3 staining; therefore, the 5% and 10% thresholds yielded identical classifications. The 15% threshold was evaluated as an exploratory sensitivity analysis.

**Supplementary Table S4.** Composition of the “other NEC” subgroup.

| **Primary site** | **n** | **POU2F3 positive, n (%)** | **Biopsy, n** | **Resection, n** |
| --- | --- | --- | --- | --- |
| Esophagus | 4 | 0 (0.0%) | 2 | 2 |
| Mediastinum | 2 | 0 (0.0%) | 1 | 1 |
| Gastrointestinal tract | 7 | 1 (14.3%) | 4 | 3 |
| Other sites | 2 | 0 (0.0%) | 1 | 1 |

The other NEC subgroup was heterogeneous and small; all analyses involving this subgroup were exploratory.

**Table S5.** Incremental diagnostic support in selected clinically relevant diagnostic scenarios.

| **Diagnostic scenario** | **n** | **POU2F3 positive, n (%)** | **Added value, n (%)** | **Supportive conventional panel, n (%)** | **Supportive with POU2F3, n (%)** | **Absolute gain, percentage points** | **Exact McNemar P** |
| --- | --- | --- | --- | --- | --- | --- | --- |
| All NEC | 140 | 28 (20.0%) | 15 (10.7%) | 113 (80.7%) | 128 (91.4%) | 10.7 | <0.001 |
| CgA-negative/low NEC | 55 | 19 (34.5%) | 13 (23.6%) | 31 (56.4%) | 44 (80.0%) | 23.6 | <0.001 |
| CgA-positive NEC | 85 | 9 (10.6%) | 2 (2.4%) | 82 (96.5%) | 84 (98.8%) | 2.4 | 0.500 |
| Marker-discordant NEC | 40 | 20 (50.0%) | 15 (37.5%) | 13 (32.5%) | 28 (70.0%) | 37.5 | <0.001 |
| Marker-concordant NEC | 100 | 8 (8.0%) | 0 (0.0%) | 100 (100.0%) | 100 (100.0%) | 0.0 | NA |

Exact McNemar tests compare paired supportive interpretations between the conventional panel and the combined panel incorporating POU2F3.

**Table S6.** Primary antibodies and immunohistochemical staining conditions used in this study.

| **Marker** | **Manufacturer** | **Clone** | **Catalog no.** | **Working concentration/dilution** | **Antigen retrieval** | **Primary incubation** | **Detection system** | **Positive control** |
| --- | --- | --- | --- | --- | --- | --- | --- | --- |
| POU2F3 | R&D Systems, Bio-Techne | 1091214 | MAB11625 | 5 μg/mL | Heat-induced epitope retrieval, basic buffer | 1 h at room temperature | HRP polymer/DAB | Known POU2F3-positive tumor |
| CgA | R&D Systems, Bio-Techne | 948128 | MAB90981 | 5 μg/mL | Laboratory-validated FFPE retrieval protocol | Overnight at 4°C | HRP-DAB | Human parathyroid or known CgA-positive NEC |
| SYN | R&D Systems, Bio-Techne | 959904 | MAB5555 | 15 μg/mL | Laboratory-validated FFPE retrieval protocol | Overnight at 4°C | HRP-DAB | Brain tissue or known SYN-positive NEC |
| CD56/NCAM1 | R&D Systems, Bio-Techne | 301021 | MAB24081 | 25 μg/mL | Heat-induced epitope retrieval, basic buffer | 1 h at room temperature | HRP polymer/DAB | Brain tissue or known CD56-positive tumor |
| INSM1 | Santa Cruz Biotechnology | A-8 | sc-271408 | 1:250 | Laboratory-validated FFPE retrieval protocol | Overnight at 4°C | HRP-DAB | Pancreatic islets or known INSM1-positive NEC |

**Figure S1.** Exploratory ROC analysis of the percentage of POU2F3-positive tumor cells for distinguishing NEC from non-NEC controls. The points corresponding to the 10% and 15% thresholds are shown.
